# The Effectiveness of Exercise as a Treatment of Major Depressive Disorder in Adolescents: A Systematic Literature Review of Randomised Control Trials

**DOI:** 10.1192/bjo.2022.226

**Published:** 2022-06-20

**Authors:** Jessica Pallot

**Affiliations:** University of Manchester, Manchester, United Kingdom

## Abstract

**Aims:**

Major depressive disorder (MDD) is the most prevalent mental health condition among adolescents. Current treatments have limited effectiveness, accessibility and questionable safety profiles. Exercise is becoming a more widely recognised intervention for MDD in adults. However, evidence and research for its effectiveness in adolescents is lacking. This review aimed to establish if exercise is effective at reducing MDD symptoms and severity in adolescents, and thus its first-line treatment potential.

**Methods:**

Electronic databases were searched for randomised control trials studying effects of exercise in adolescents, clinically diagnosed with MDD. Trials were excluded if participants’ depression was secondary to another disorder or health condition. The primary outcome measure was depression symptom severity, assessed by a validated depression symptom scale. Six trials met the eligibility criteria and were included in this review.

**Results:**

Four trials found reduced depression scores in the exercise intervention group compared to control immediately post-intervention; of the four trials which included follow-up data, all reported higher rates of remission in the exercise intervention group compared to control. The length of exercise intervention programme seems important, needing to be greater than 6-weeks for a therapeutic effect. The type of exercise doesn't appear critical.

**Conclusion:**

Given the small sample sizes and methodological limitations presented by the trials, it is difficult to draw definitive conclusions. Further and larger-scale studies are needed before exercise can become a recognised and readily recommended treatment for MDD in adolescents; but thus far, it seems to have a promising therapeutic potential in both short and long term.

